# Transepithelial Phototherapeutic Keratectomy Using a 213-nm Solid-State Laser System Followed by Corneal Collagen Cross-Linking with Riboflavin and UVA Irradiation

**DOI:** 10.1155/2010/146543

**Published:** 2010-11-02

**Authors:** George D. Kymionis, Michael A. Grentzelos, Alexandra E. Karavitaki, George A. Kounis, Georgios A. Kontadakis, Sonia Yoo, Ioannis G. Pallikaris

**Affiliations:** ^1^Institute of Vision and Optics, Faculty of Medicine, University of Crete, 71003 Heraklion, Crete, Greece; ^2^Bascom Palmer Eye Institute, University of Miami, Miami, FL 33136, USA

## Abstract

*Purpose*. To present a case of a keratoconic patient who underwent epithelial removal with transepithelial phototherapeutic keratectomy (t-PTK) using a 213-nm solid-state laser system followed by corneal collagen cross-linking (CXL) with riboflavin and ultraviolet-A (UVA) irradiation. *Methods*. Case report. *Results*. A twenty-four-year-old male with keratoconus underwent CXL treatment after epithelial removal with t-PTK using a solid-state laser system. No intra- or early postoperative complications were found. One month postoperatively, uncorrected visual acuity (UCVA) improved from 20/63 to 20/32 while best spectacle- corrected visual acuity (BSCVA) improved from 20/40 to 20/25. Corneal topography revealed a significant improvement which remained stable during the six-month followup period. *Conclusions*. Epithelial removal with t-PTK before CXL could improve patient's visual outcome.

## 1. Introduction

Removal of the corneal epithelium is an essential component of riboflavin-ultraviolet-A (UVA) corneal collagen cross-linking (CXL) therapy to permit the penetration of riboflavin into the corneal stroma. Failure to achieve adequate stromal absorption of riboflavin may impair the efficacy of the cross-linking process [1]; until now epithelial removal is performed mechanically. Excimer laser phototherapeutic keratectomy (PTK) is a well-known surgical technique that has successfully been used in the management of superficial corneal pathology, such as anterior corneal dystrophies, degenerations [2], and the treatment of keratoconus nodules [3]. Transepithelial PTK (t-PTK) uses an Excimer laser ablation in order to remove the epithelium and smooth the anterior irregular cornea [4].

In this paper, we present a case of a keratoconic patient who underwent epithelial removal with t-PTK using a solid-state laser system followed by CXL treatment with riboflavin and UVA irradiation.

## 2. Case Report

A twenty-four-year-old male with progressive keratoconus presented to our institute for consultation. At the time of the examination, uncorrected visual acuity (UCVA) was 20/25 in the right and 20/63 in the left eye and best spectacle-corrected visual acuity (BSCVA) was 20/20 (manifest refraction plano—1.00 × 15) in the right eye and 20/40 (manifest refraction—1.25 – 4.00 × 125) in the left eye. A trial with a rigid gas-permeable contact lens demonstrated improvement in visual acuity of 20/20 in his left eye. Keratometric readings were 42.60 × 24/43.52 × 114 at the right and 47.51 × 46/ 56.98 × 136 at the left eye.

In order to assess corneal thickness, a pachymetric map measurement was performed in the right (481 *μ*m central, 639 nasal, 550 temporal, 587 superior, and 547 inferior) and left (462 *μ*m central, 581 nasal, 569 temporal, 611 superior, and 541 inferior) eye by ultrasound pachymetry (Sonogage, Cleveland, Ohio, USA). Epithelial thickness at the apex of the cone was 41 *μ*m in the left eye. Slit-lamp examination of anterior and posterior segment showed no other abnormality.

### 2.1. Surgical Technique

**Figure 1:**
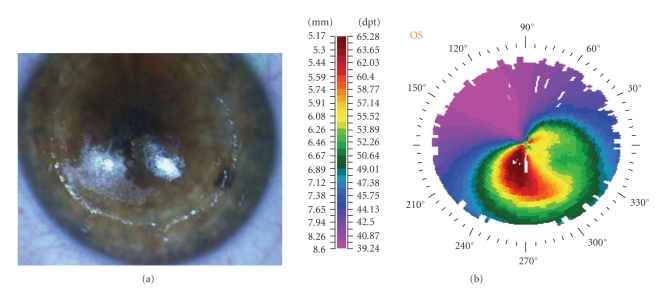
Intraoperative photography (a) showing the correlation between the ablated stroma (coarse surface) after transepithelial phototherapeutic keratectomy (t-PTK) and the preoperative corneal topography (b).

Epithelium was removed by t-PTK. A solid-state laser system with a wavelength of 213 nm (Pulzar Z1, CustomVis, Perth, WA) was used for the procedure. The wavelength is generated using a major Nd:YAG laser system of 1064 nm, and through special cultivated crystals the 213 nm is finally used. The t-PTK ablation was performed in an 8.0 mm zone in an intended depth of 50 *μ*m. There was a correlation between the localized ablated stroma and the preoperative topography (Figure 1). After t-PTK, riboflavin 0.1% solution was instilled every 3 minutes for approximately 30 minutes. Penetration of the cornea and presence of riboflavin in the anterior chamber (riboflavin shielding) were monitored by slit-lamp examination. UVA irradiation was performed using a commercially available UVA optical system (UV-X illumination system version 1000, Zurich, Switzerland) with a light source consisting of an array of UV diodes (365 nm) with a potentiometer in series to allow regulation of voltage. Before treatment, intended irradiance of 3.0 mW/cm^2^ (5.4 J/cm^2^ surface dose after 30 minutes) was calibrated using the UV-A light meter YK-34UV (Lutron Electronic) which is supplied with the UV-X device. Irradiance was performed for 30 minutes, corresponding to a dose of 5.4 J/cm^2^. During treatment, riboflavin solution was applied every 5 minutes to saturate the cornea with riboflavin. At the end of the procedure, a silicon-hydrogel bandage contact lens (Lotrafilcon B, Air Optix, Ciba Vision—14.0 mm diameter, 8.6 base curvature, and Dk = 140 barrers) was applied until full reepithelialization.

Postoperative medication included diclofenac sodium 0.1% (Denaclof, Novartis) for 2 days as well as antibiotic/corticosteroid (tobramycin/dexamethasone) drops (Tobradex, Alcon Laboratories, Inc.) four times daily until the removal of the bandage contact lens. The treated left eye was examined daily until the epithelium was completely healed. Contact lens was removed at the fifth postoperative day, and no signs of edema or inflammation were cited by slit-lamp biomicroscopy. After the removal of the contact lens, the patient received corticosteroid drops (FML, fluorometholone 0.1%, Falcon Pharmaceuticals), tapering for the next 15 days, and he was encouraged to use artificial tears at least six times per day for 3 months postoperatively. 

**Figure 2:**
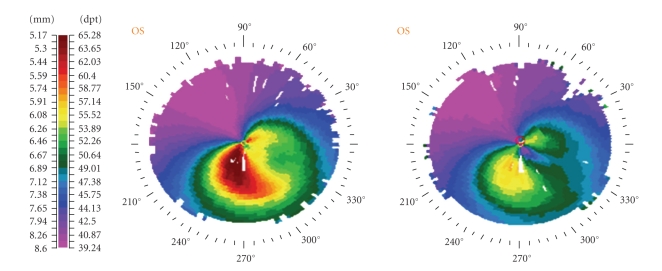
Preoperative (left) and one-month postoperative (right) topography (Technomed C-Scan, Baesweiler, Germany) of the keratoconic patient treated with combined transepithelial phototherapeutic keratectomy and collagen cross-linking with riboflavin and UVA irradiation.

 One month after the combined t-PTK and CXL procedure, both UCVA and BSCVA were improved at 20/32 and 20/25, respectively (manifest refraction —0.50 – 2.00 × 135). Slit-lamp biomicroscopy revealed a clear cornea with no signs of haze formation. Corneal topography revealed a significant improvement (Figure 2) which remained stable during the six-month follow-up period.

## 3. Discussion

Excimer laser phototherapeutic keratectomy (PTK) is a well-known surgical technique that has successfully been used in the management of superficial corneal pathology, such as anterior corneal dystrophies and degenerations [2]. Elsahn et al. demonstrated the safety and efficacy of PTK in the treatment of keratoconus nodules and the improvement of contact lens tolerance in these patients [3]. T-PTK uses an Excimer laser ablation to remove the epithelium and smooth the anterior irregular cornea [4].

In this paper, the patient underwent t-PTK using a solid-state laser system before CXL with riboflavin and UVA irradiation. The aim of t-PTK was epithelial removal and anterior cornea smoothening in order to decrease the irregular astigmatism. In keratoconic patients, epithelium is thinner at the cone apex than centrally [5]. Reinstein has reported that in a study of 38 keratoconic eyes, an inferotemporal region of thin epithelium surrounded by epithelial thickening was found; a pattern coincided with the cone on topography [6]. According to this keratoconic pattern, an intended ablation depth of 50 *μ*m has been chosen in order to use the patient's own epithelium as a masking agent allowing laser ablation to take place on the apex of the cone to regularize the stromal surface (Figure 1). 

The significant improvement in visual acuity, especially in UCVA, from the first postoperative month should be the result of t-PTK which decreases corneal irregularities as shown at the topography one month postoperatively (Figure 2). CXL after mechanical epithelial removal could potentially improve patient's UCVA but not at the first postoperative month and in this amplitude [7]. A possible limitation of this approach could be the decrease of corneal tissue by t-PTK at the cone apex before CXL. This could increase the possibility of corneal damage from the UVA irradiation.

In conclusion, epithelial removal with t-PTK before CXL could improve patient's visual outcomes. Longer follow-up is necessary in order to evaluate the outcomes of this combined procedure.
